# Epithelioid Angiosarcoma of the Septum Pellucidum

**DOI:** 10.1155/2013/603671

**Published:** 2013-09-19

**Authors:** Chiara Baldovini, Matteo Martinoni, Gianluca Marucci

**Affiliations:** ^1^Section of Pathology M. Malpighi at Bellaria Hospital, Department of Biomedical and Neuro Motor Sciences, University of Bologna, Via Altura 3, 40139 Bologna, Italy; ^2^Department of Neurosurgey, IRCCS Institute of Neurological Science, Bellaria Hospital, Via Altura 3, 40139 Bologna, Italy

## Abstract

Primary cerebral intra-axial epithelioid angiosarcoma is an extremely rare malignancy. To the best of our knowledge we describe the first case of epithelioid angiosarcoma arisen in the septum pellucidum of a 54-years-old man. Albeit extremely rare, this neoplasia is a potential source of misdiagnosis for other aggressive malignant tumors, and it should be taken into consideration.

## 1. Introduction

Primary epithelioid angiosarcoma of the central nervous system (CNS) is an extremely rare malignancy; only one case was reported in literature showing immunoreactivity to epithelial markers [1]. Here, we describe, to the best of our knowledge, the first case of epithelioid angiosarcoma arising from the septum pellucidum. 

## 2. Case Report

The patient was a 54-years-old man presenting with severe headache and vomiting that lasted for three days. He showed signs of mental confusion and progressively worsening neurological symptoms. A CT scan performed in the emergency department showed a triventricular hydrocephalus associated with a solitary hemorrhagic lesion involving the septum pellucidum. The patient was treated with an external ventricular drainage that resulted in a prompt improvement of the clinical symptoms. A few days later, an MRI scan confirmed the presence of an inhomogeneous mass both in T1 and T2 WI, showing a strong enhancement after gadolinium injection. The lesion extended also into the third ventricle, thus causing a severe stenosis of both Monro's foramina. 

The patient was submitted to a right frontal craniotomy and, through a transcallosal approach, to a gross total excision of the bleeding neoplasm. Furthermore, a wide septostomy was performed, leaving the left external drainage in place. During postsurgical course, a total body-CT scan excluded the presence of neoplastic lesions other than that in the CNS. The tumor showed a rapid regrowth, and repeated further bleedings with consequent ventricular dilation, which required a right ventricular external drainage. About two months after surgery, the patient deceased, but an autoptic examination could not be performed.

**Figure 1:**
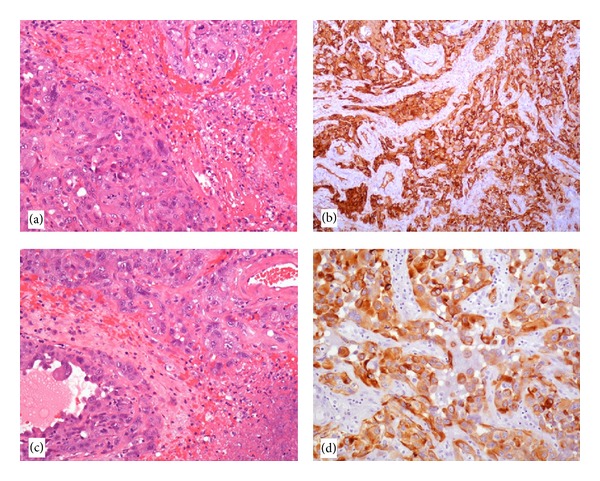
The tumor is composed of pleomorphic, epithelioid cells with prominent nucleoli and atypical mitotic figures (a) and necrotic areas (b) (hematoxylin and eosin, 200x magnification). Neoplastic cells are strongly positive for CD31 (c) (100x magnification) and cytokeratin (d) (200x magnification).

The histological examination highlighted an extensively necrotic and hemorrhagic neoplasms composed of large, pleomorphic, round to polygonal epithelioid cells, with vesicular and central to eccentrically located nuclei, prominent nucleoli and abundant eosinophilic cytoplasm (Figures 1(a) and 1(b)). Neoplastic cells were mainly arranged in sheets and nests, but tubular structures were occasionally seen. Moreover, irregularly shaped thick-walled vessels were present, lined by atypical epithelioid cells. Mitotic figures were numerous, sometimes atypical, and Ki-67 labelling index was very high. The tumour was strongly positive for CD31 (Figure 1(c)), factor VIII and cytokeratin MNF116 (Figure 1(d)) and negative for glial fibrillary acidic protein (GFAP), epithelial membrane antigen (EMA), Mart-1, and S-100 protein. These findings are consistent with a diagnosis of epithelioid angiosarcoma.

The occurrence of a cerebral epithelioid angiosarcoma represents a rare event; however, several entities should be considered in the differential diagnosis. In this case, the location of the lesion and its histological epithelioid appearance suggested to take into account a diagnosis of anaplastic ependymoma. Further, ependymomas may be immunopositive for cytokeratins [2]. However, the complete absence of GFAP and EMA ruled out this diagnosis. Glioblastoma (GBM) can show high cellularity, nuclear polymorphism, increased mitotic activity, stromal areas with a desmoplastic to a fibromyxoid appearance, and, obviously, necrosis and vascular proliferation. Furthermore, GBM may display sarcomatous changes (gliosarcoma), with vascular [3] differentiation. However, the immunopositivity for GFAP and the absence of immunoreactivity with vascular antibodies, typically observed in GBM, permitted a prompt distinction. Moreover, the highly malignant appearance suggested to include metastatic melanoma in the differential diagnosis, but the lack of expression of melanocytic markers (MART-1 and S-100) in our case indicated that this hypothesis could be rejected. Finally, considering the immunopositivity for cytokeratins, a choroid plexus carcinoma or a metastatic carcinoma had to be ruled out. Both these entities were excluded with the help of immunohistochemistry; furthermore, choroid plexus carcinoma occurs usually in children.

As previously stated, only one case of primitive cerebral epithelioid angiosarcoma expressing epithelial markers has been published in the literature [1]: the neoplasm was located in the right parietal lobe of a 39-year-old man, who died 29 months after the clinical presentation. Interestingly, Lach and colleagues [4] described one case of primary composite leiomyosarcoma-epithelioid angiosarcoma of the frontoparietal region of a 37-year-old man. The epithelioid component was focally positive for factor VIII and CD31, but lacked immunohistochemical reactivity for cytokeratins AE1–AE3 and CAM5.2. Moreover, epithelioid features had been focally described in 50% of cases of the angiosarcoma of the brain presented by Mena et al. [5], although there was no mention of immunoreactivity to epithelial markers. 

## 3. Conclusions

In conclusion, cerebral primitive epithelioid angiosarcoma, albeit extremely rare, could be considered as a potential source of misdiagnosis for other aggressive malignant tumors of the CNS.
